# Supplementary material for: TRPV1 regulates excitatory innervation of OLM neurons in the hippocampus
Source: Nat Commun. 2017 Jul 19;8:15878. doi: 10.1038/ncomms15878 (PMC5524938; doi:10.1038/ncomms15878)
Supplement: Supplementary Information [file ncomms15878-s1.pdf]

Type of file: pdf
Size of file: 0 KB
Title of file for HTML: Supplementary Information
Description: Supplementary Figures

Type of file: pdf
Size of file: 0 KB
Title of file for HTML: Peer Review File
Description:

Supplementary Figure 1

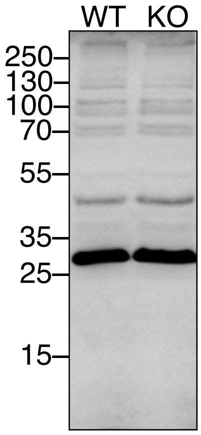

Supplementary Figure 1. C-terminal TRPV1 antibodies do not recognize a TRPV1 band of the correct size. Western blot showing that the C-T TRPV1 antibody (from Millipore) does not recognize a specific band at the expected size for TRPV1 in WT and TRPV1 KO homogenates from whole brain.

Supplementary Figure 2

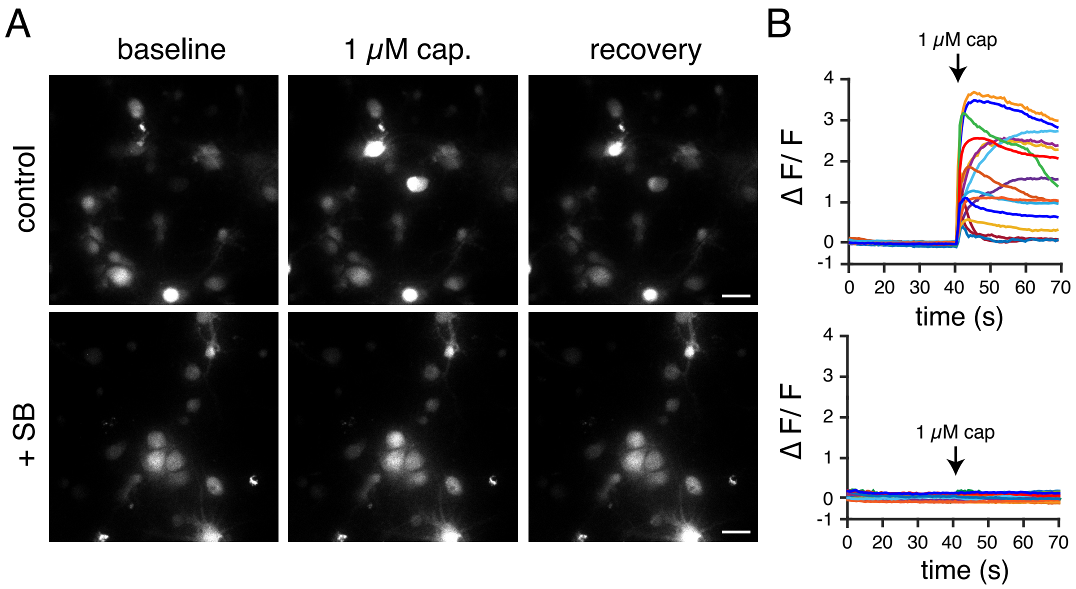

Supplementary Figure 2. Calcium influx is induced by capsaicin in a subset of hippocampal neurons, and is blocked by the TRPV1 antagonist SB-366791. A) Representative Fluo-4-based Ca^{2+} images in dissociated rat hippocampal cultures 30 s before, during and 30 s after 1 μM capsaicin stimulation in control conditions, and in the presence of 1 μM concentration of the TRPV1 blocker SB-366791; scale bar = 20 μm. **B)** Representative traces of Fluo-4 signal in control (upper panel) and 1 μM SB-366791 treated (lower panel) conditions indicate that a sub-population of hippocampal neurons in control cultures responds to 1 μM capsaicin treatment (arrow), and responses are abolished in the presence of the TRPV1 blocker SB-366791 (n=40-45 time-lapse recordings; 3 different cultures; 23 of 574 cells responded to capsaicin in control conditions, and 1 of 545 cells responded to capsaicin in the presence of SB-366791).

Supplementary Figure 3

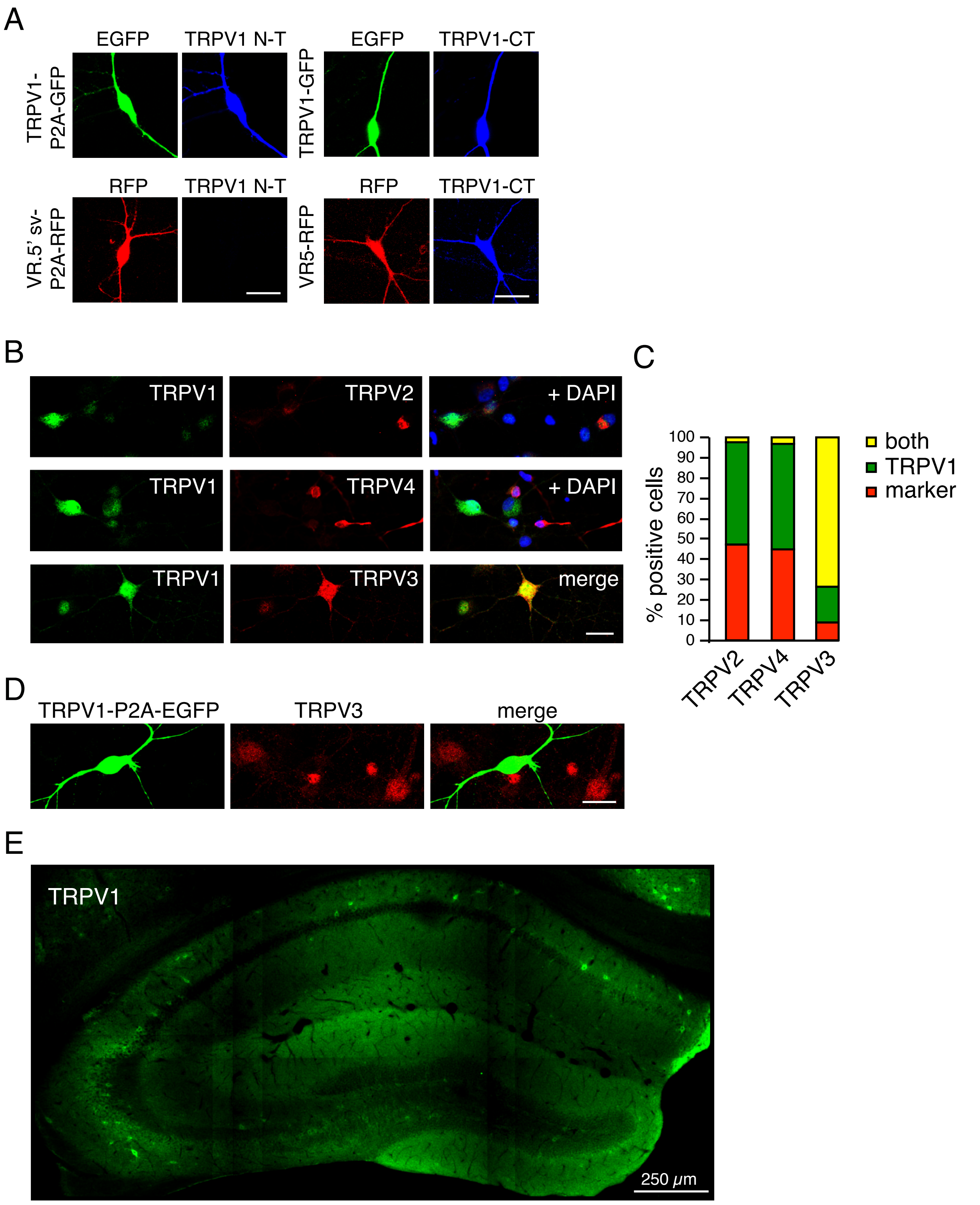

Supplementary Figure 3. The C-terminal TRPV1 antibody recognizes VR.5' sv, but not TRPV2, TRPV3, or TRPV4. A) Both the N-terminal and C-terminal TRPV1 antibodies detect mouse hippocampal neurons transfected with TRPV1-P2A-EGFP, but only the C-terminal TRPV1 antibody detects neurons transfected with VR.5' sv-P2A-RFP; scale bar = 20 μm. **B)** Immunostains of hippocampal neurons with TRPV1 and TRPV2, TRPV3, or TRPV4 antibodies. The C-terminal TRPV1 antibody does not recognize the homologous TRPV2 or TRPV4 channels, which are expressed in different subsets of mouse hippocampal neurons *in vitro*, but TRPV1 and TRPV3 signals colocalize in a subset of neurons, quantified in **(C)** (n=20 images each; 2 different cultures; scale bar = 20 μm)**. D)** The TRPV3 antibody does not recognize overexpressed TRPV1 in hippocampal neurons; scale bar = 20 μm.

Supplementary Figure 4

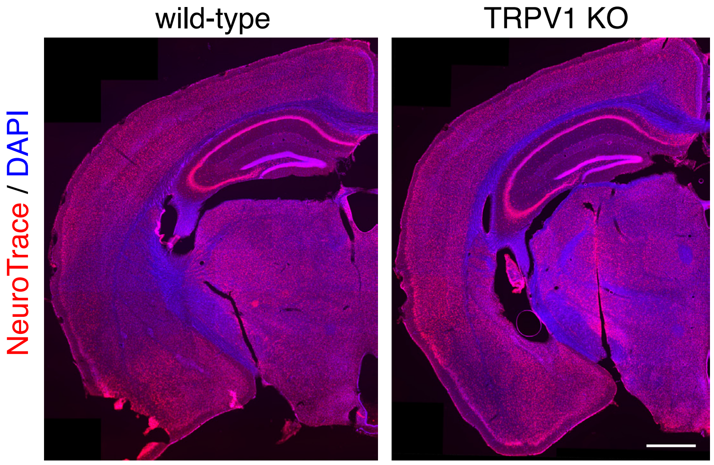

Supplementary Figure 4. TRPV1 knockout mice have normal cortical and hippocampal laminar architecture. **A)** Immunostains of brain sections with Neurotrace and DAPI showed no obvious alteration in gross morphology of the adult brain in TRPV1 knockouts compared to wild-type mice (scale bar = 1 mm).

Supplementary Figure 5

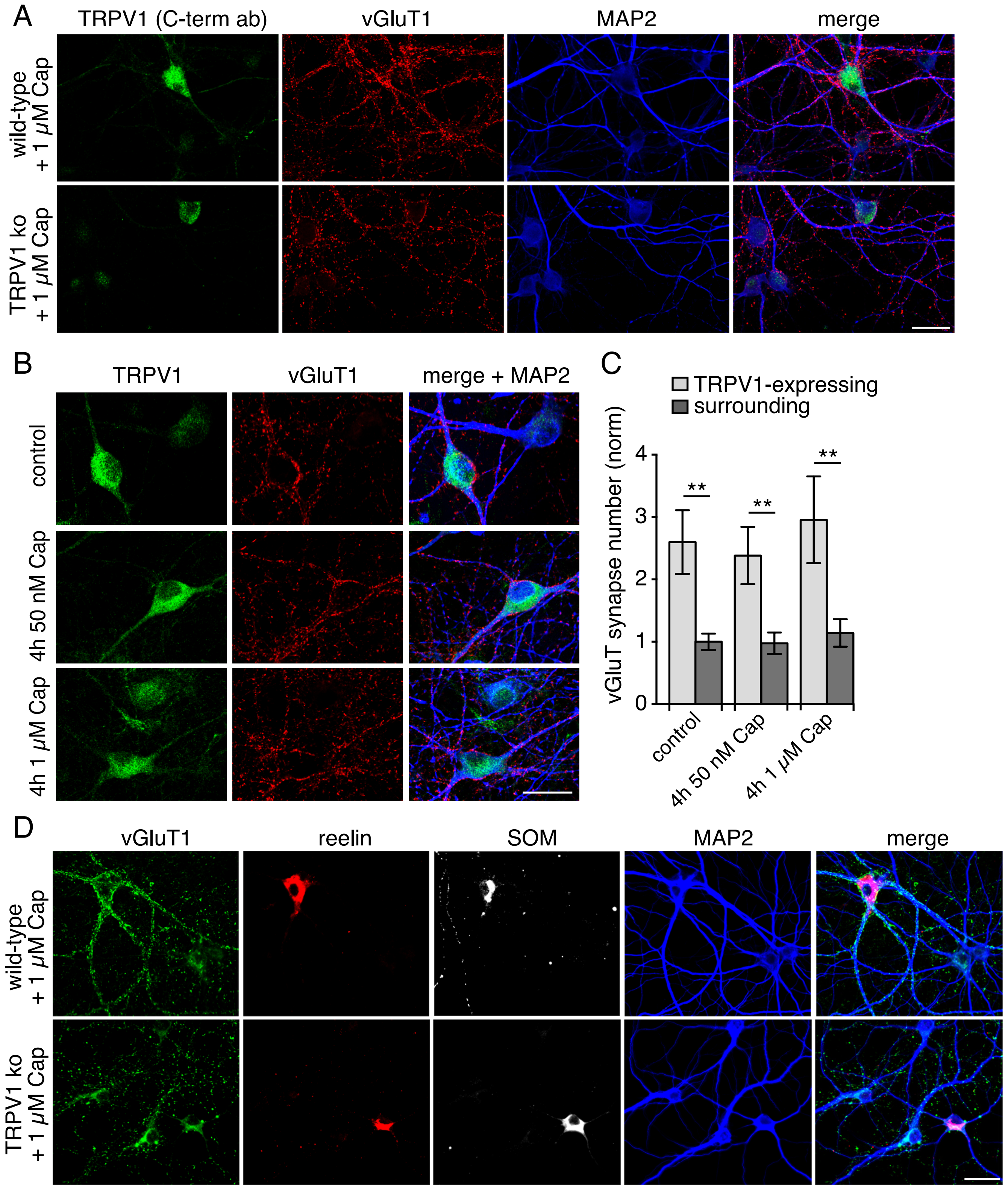

Supplementary Figure 5. **Capsaicin treatment for only 4 hours does not further increase excitatory innervation of TRPV1-expressing neurons. A)** Images of wild-type and TRPV1 knockout neurons immunostained with the C-terminal TRPV1 antibody (which recognizes a remaining splice isoform in the knockouts), vGluT1, and MAP2 following overnight treatment with 1 μM capsaicin, showing both TRPV1-expressing neurons and surrounding non-TRPV1-expressing neurons. **B)** Images of hippocampal neurons immunostained for TRPV1, vGluT1, and MAP2 in control conditions, and following treatment with 50 nM or 1 μM capsaicin for 4 hours. **C**) Quantitation of excitatory synapse number (number of vGluT1-positive puncta) on TRPV1-positive hippocampal neurons normalized to surrounding cells in the indicated conditions. Images used for quantitation were: control n=12, 50nM cap. n=12, 1 μM cap. n=9; from 3-4 cultures. Error = SEM; significance determined by one way ANOVA with Tukey's post hoc test for multiple comparisons. **D)** Images of wild-type and TRPV1 knockout neurons immunostained with vGluT1, reelin, SOM and MAP2 following overnight treatment with 1 μM capsaicin, showing both TRPV1-expressing neurons and surrounding non-TRPV1-expressing neurons; merge indicates combination of vGluT1, reelin and MAP2 channels; scale bars = 20 μm in all panels.

Supplementary Figure 6

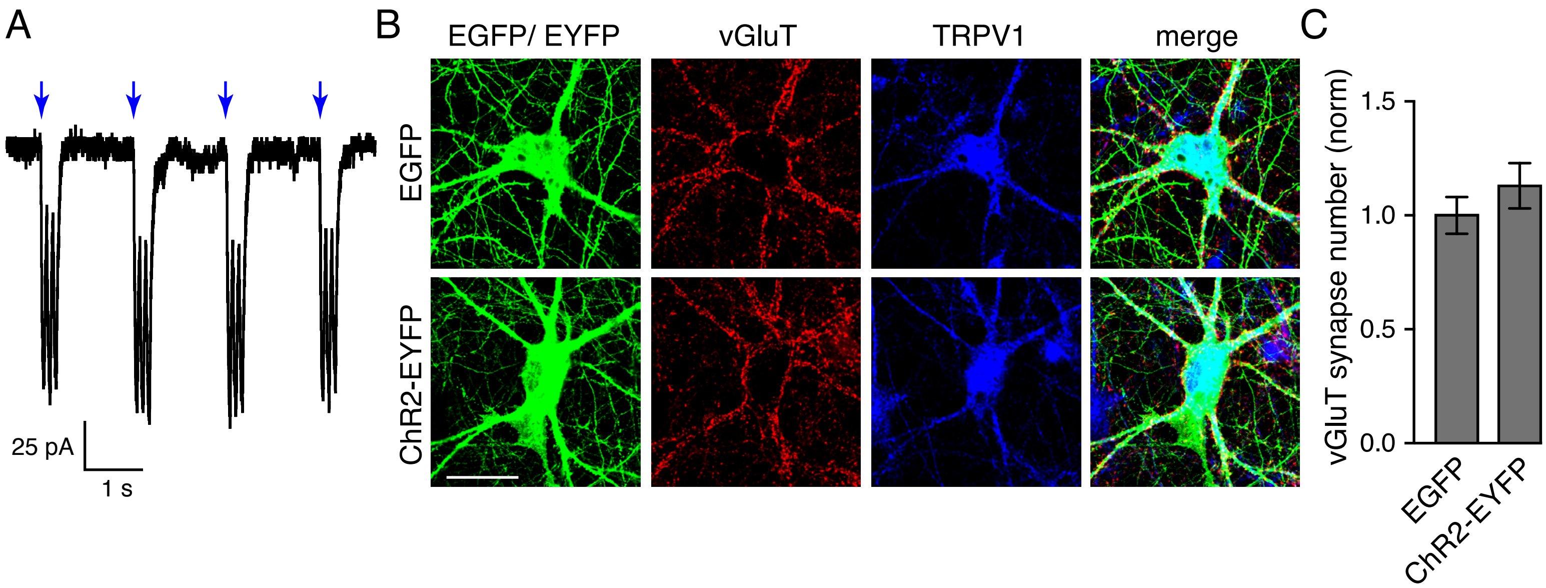

Supplementary Figure 6. Increasing activity by optogenetic stimulation does not increase excitatory innervation of TRPV1-expressing neurons. A) Sample trace of a whole cell patch clamp recording from a ChR2-EYFP transduced dissociated hippocampal neuron culture stimulated with blue light by LED. **B)** Immunostains of EGFP or EYFP signal, vGluT and TRPV1 in EGFP AAV and ChR2-EYFP transduced neurons following optogenetic stimulation with blue light for 24 hours; scale bar = 20 μm. **C)** Quantitation of excitatory synapse number on TRPV1-expressing neurons in the indicated conditions following stimulation (n = 28 neurons/ images for each condition from 4 different cultures; error = SEM, significance determined by unpaired Student's t-test with Welch's correction).

Supplementary Figure 7

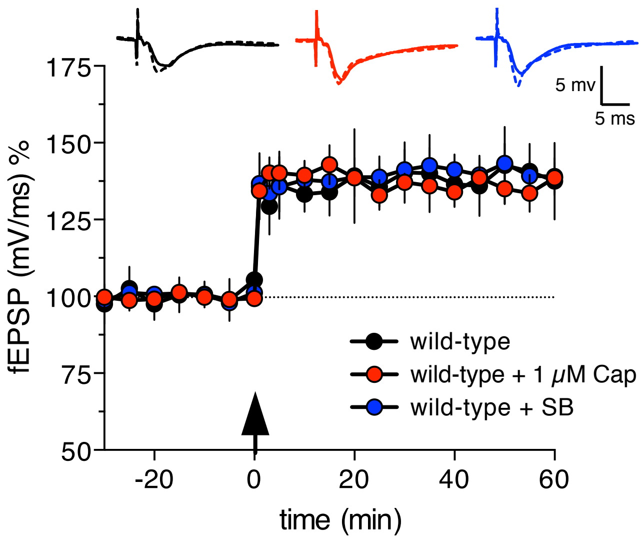

Supplementary Figure 7. Acute activation or blockade of TRPV1 does not affect Schaffer collateral LTP. LTP induced by 1XTET-LTP in the Schaffer collateral pathway in wild-type hippocampal slices treated with 1 μM capsaicin, or with 1 μM SB-366791 to block TRPV1 channels, 30 minutes prior to and during LTP, compared to controls (n = 7 slices/ mice for each condition; significance determined by Student's t-test, error = SEM). Representative fEPSP traces 30 minutes before (solid line) and 60 minutes after (dashed line) LTP induction are shown above LTP traces.
